# An environmental transfer hub for multimodal atom probe tomography

**DOI:** 10.1186/s40679-017-0045-2

**Published:** 2017-05-02

**Authors:** Daniel E. Perea, Stephan S. A. Gerstl, Jackson Chin, Blake Hirschi, James. E. Evans

**Affiliations:** 1 Environmental Molecular Sciences Laboratory, Pacific Northwest National Laboratory, 3335 Innovation Boulevard, Richland, WA 99354 USA; 2 Scientific Center for Optical and Electron Microscopy (ScopeM), ETH Zurich, Auguste-Piccard-Hof 1, Zurich, 8093 Switzerland

**Keywords:** Atom probe tomography, Environmental transfer, Cryogenic transfer, Cryo atom probe tomography

## Abstract

Environmental control during transfer between instruments is required for samples sensitive to air or thermal exposure to prevent morphological or chemical changes prior to analysis. Atom probe tomography is a rapidly expanding technique for three-dimensional structural and chemical analysis, but commercial instruments remain limited to loading specimens under ambient conditions. In this study, we describe a multifunctional environmental transfer hub allowing controlled cryogenic or room-temperature transfer of specimens under atmospheric or vacuum pressure conditions between an atom probe and other instruments or reaction chambers. The utility of the environmental transfer hub is demonstrated through the acquisition of previously unavailable mass spectral analysis of an intact organic molecule made possible via controlled cryogenic transfer into the atom probe using the hub. The ability to prepare and transfer specimens in precise environments promises a means to access new science across many disciplines from untainted samples and allow downstream time-resolved in situ atom probe studies.

## Background

Atom probe tomography (APT) is a powerful technique that is able to determine both the three-dimensional (3D) structure and elemental composition of materials with atomic resolution across many disciplines [1–9] APT analysis commonly involves the use of a dual beam scanning electron microscope/focused ion beam (FIB/SEM) [10] to prepare samples in a compatible geometry. Moving prepared specimens from the FIB/SEM to the atom probe typically requires the specimen to be transferred between instruments under ambient conditions. However, for specimens that are sensitive to air (i.e., oxygen, water, etc.) or thermal exposure, it is imperative to insure environmental protection during the entire transfer process so that all specimens are maintained in a pristine state before, during, and after analysis. The first part, before analysis, is critical for future cryogenic APT developments enabling broader applications in biology, polymer, and organic chemistry. The latter part, after analysis, is critical for future time-resolved studies such as APT analysis of nanoscale catalyst materials in one state then undergoing oxidation/reduction and then analyzing the same sample in a second state.

One approach for controlled transfer is to utilize a shuttle device, a *suitcase* if you will, in which specimens are transported between the preparation instrument(s) and the analysis instrument under a well-defined environment (e.g., user-set temperature in inert gas or high vacuum). Accomplishing this task requires that all tools involved have a compatible docking port for such a shuttle, as well as be able to accept and manipulate the same specimen holder. While such a system works [6, 11], it is not conducive with research settings where the multiple instruments are from various vendors and may not have compatible docking ports or even the ability to accept a common shuttle or manipulate the same specimen holder geometry. The other approach would be to develop a centralized system that allows for environmental transfer into the APT and docking with various holder geometries, shuttles, reaction chambers, etc.

In this article, we describe a novel system consisting of an environmental transfer hub station (ETH) compatible with a customized specimen shuttle device that allows the environmentally controlled transfer of specimens between a FIB/SEM and a local electrode atom probe (LEAP). The utility of this capability is demonstrated through the cryo-preparation, transfer, and APT analysis of a deposited film of an organometallic molecule facilitated by the ETH. Such versatile and controlled transfer provided new pristine mass spectra to be recorded and promises to allow one to access new complex nanoscale biological and materials science that cannot be accessed otherwise.

**Fig. 1:**
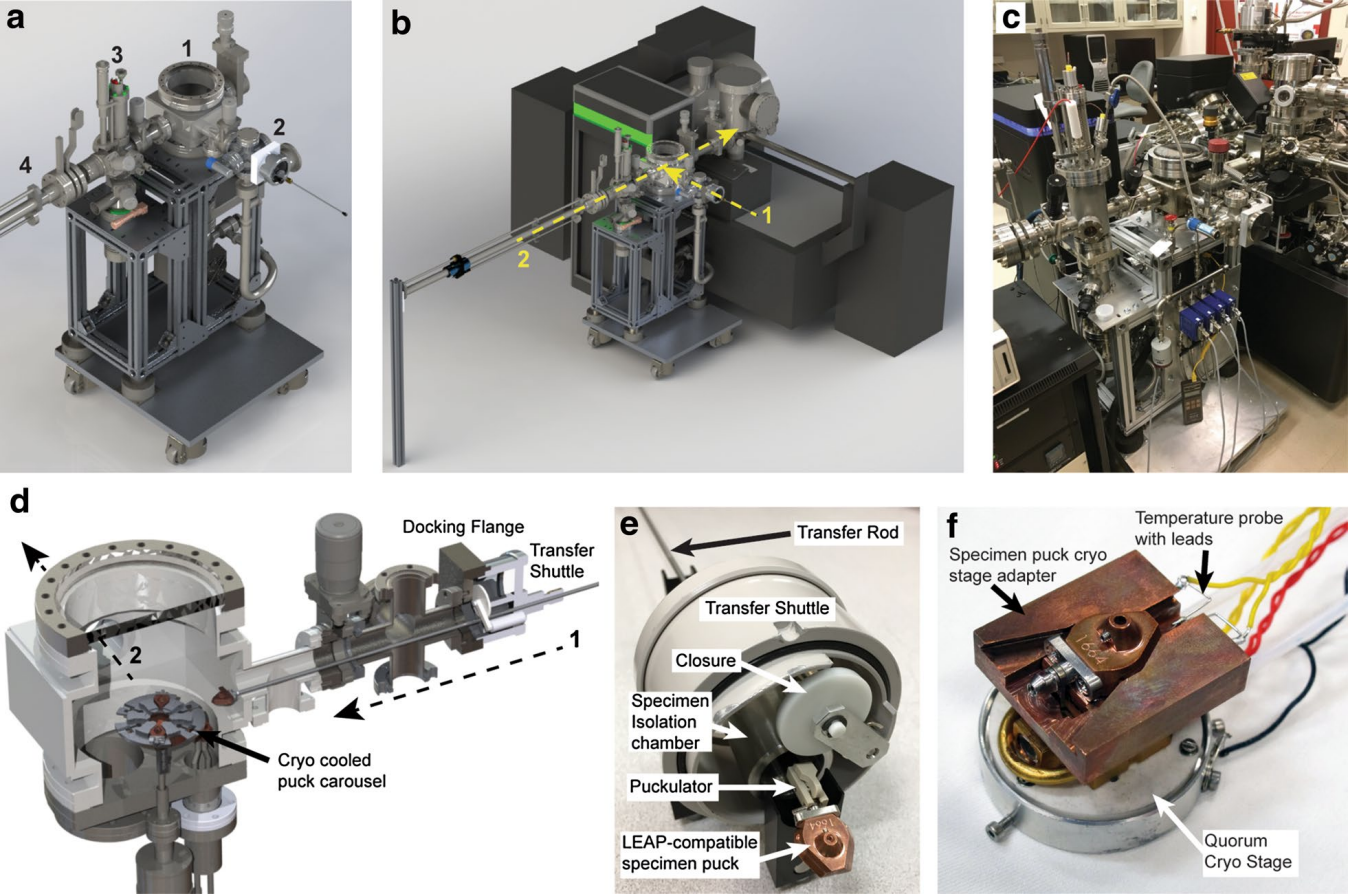
The ETH design and configuration. **a** CAD-rendered image of the ETH isolated from the local electrode atom probe (LEAP) with the various main parts as (*1*) main vacuum chamber hub, (*2*) docking port for specimen transfer shuttle (shown connected), (*3*) high-temperature ambient pressure reactor chamber, (*4*) manipulator transfer specimens between the ETH and the LEAP. **b** CAD-rendered view of the ETH connected to the LEAP. *Arrow 1* indicates transfer of the specimen from the specimen shuttle to the ETH. *Arrow 2* indicates the transfer of the specimen from the ETH to the LEAP buffer chamber. **c** Photograph of the ETH system connected to the LEAP at PNNL. **d** CAD-rendered image of the ETH main vacuum hub with the specimen shuttle docked. The shuttle transfer rod with LEAP specimen puck is extended toward a cryo-cooled puck carousel. The *dashed arrows* correlate with the *yellow dashed arrows* in (**b**). **e**–**f** Photographs of the environmental transfer shuttle and FIB/SEM cold stages, respectively

**Fig. 2:**
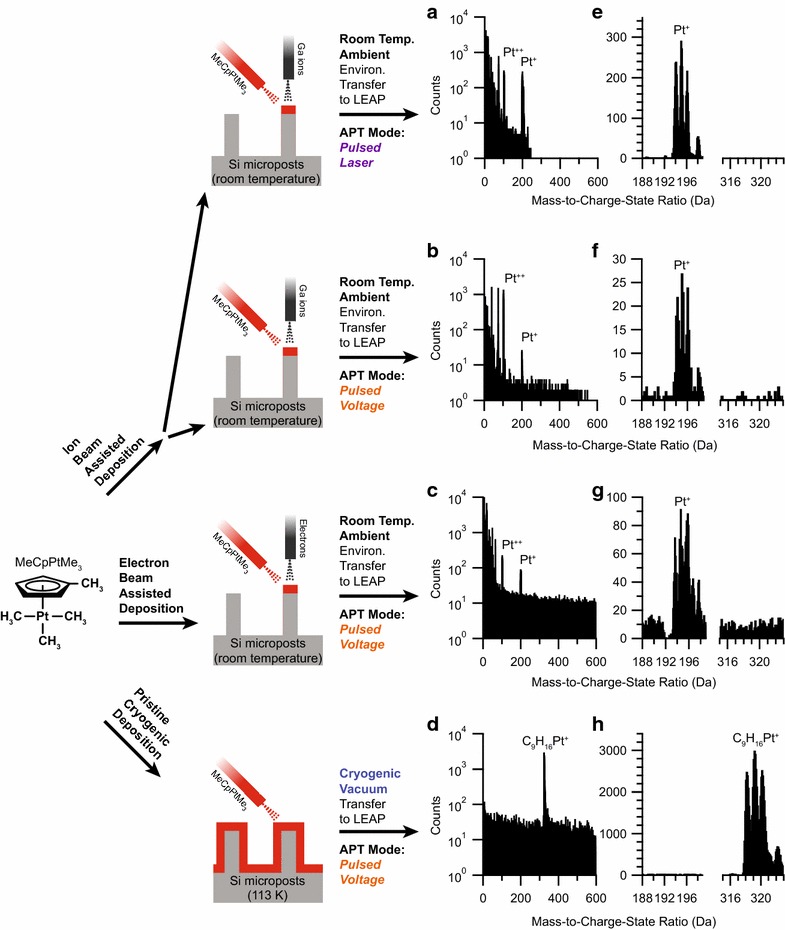
Specimen preparation and APT ToF mass spectra of MeCpPtMe_3_ prepared under ambient and cryogenic conditions. **a** Mass spectrum of MeCpPtMe_3_ decomposed at room temperature into Pt/a-C via IBAD and collected via (**a**) APT laser-pulsed mode and **b** APT voltage-pulsed mode. **c** Mass spectrum of MeCpPtMe_3_ decomposed at room temperature into Pt/a-C via EBAD and collected via APT-voltage-pulsed mode. **d** Mass spectrum collected via APT voltage-pulsed mode of MeCpPtMe_3_ deposited onto a Si micropost array substrate cryogenically cooled to 113 K in the FIB/SEM and environmentally transferred under high vacuum into the atom probe. **e**–**h** Zoomed in region of mass spectra showing individual isotope mass peaks of Pt

**Fig. 3:**
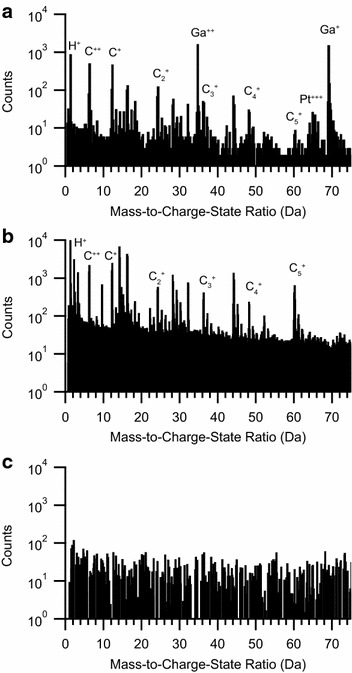
Mass spectral analysis of carbon and related species. Selected region of mass spectrum showing mass peaks for elemental carbon and complex carbon ions from a specimen prepared from MeCpPtMe_3_ at room temperature via **a** IBAD and **b** EBAD. Note the presence of Ga in (**a**) as it was prepared by IBAD. **c** The mass spectrum from a cryogenically prepared and environmentally transferred specimen of MeCpPtMe_3_ does not show evidence of elemental carbon or complex carbon ion mass peaks

The ETH consists of a central cuboid-shaped ultrahigh vacuum (UHV) chamber (Fig. 1a, 1) with connection ports to a customized specimen transfer shuttle docking port (Fig. 1a, 2) for vacuum or cryogenic transfer and a high-temperature ambient pressure reactor system (Fig. 1a, 3) for transfer under controlled atmospheres. In either case, specimens are transferred between the ETH central chamber and the LEAP via the specimen transfer rod (Fig. 1a, 4). Note that a detailed description of the add-on high-temperature ambient pressure reactor system and controlled atmosphere transfer will be the subject of a separate manuscript. A turbo pump, not shown in the view of Fig. 1, is used to maintain a base pressure of <5 × 10^−6^ Pa. The ETH is maintained by an aluminum frame supported by passive vibration isolation mounts, all of which are supported by an aluminum plate with four leveling caster wheels. The ETH interfaces directly to the front 4 ½ in. UHV buffer chamber flange of the LEAP via a bellows-style coupling tube, the configuration and orientation of which are shown in Fig. 1b. The design of the ETH is such that it can easily be decoupled from the LEAP and moved to allow for an unobstructed access to the LEAP if needed for maintenance or repair. In addition, the vibration isolation mounts and bellows-style coupling minimize vibration to the LEAP system, and thus either system can be operated independently without interference from each other. The completed ETH system is shown in Fig. 1c attached to the LEAP 4000 system housed at PNNL.

The environmental transfer of specimens between the FIB/SEM (used for specimen processing) and the LEAP (used for analysis) is facilitated via a docking flange and an environmental transfer shuttle (Fig. 1d, e). Modifications were made to the Quorum PT2010T shuttle device to accommodate the handling of LEAP specimen pucks (Fig. 1e). The cold stage for the Quorum PT2010T system is shown in Fig. 1f, and mounts within the FIB/SEM chamber. In order to accommodate LEAP specimen pucks, a cryo-stage adapter with a dovetail fitting was fabricated from Cu that attaches directly to the cryo-stage. The stage adapter also has an integrated temperature probe that interfaces with the existing Quorum system to precisely measure and control temperature over a range from about 370 K down to 100 K or lower. To minimize thermal transfer, a customized effector (puckulator) was fabricated from polychlorotrifluoroethylene plastic (Kel-F) which secures the LEAP puck for manipulation. Prior to the undocking of the shuttle for transport between the FIB/SEM and the LEAP, the specimen is retracted into a chamber isolated with a closure under HV conditions or backfilled to near atmospheric pressure under an inert atmosphere (e.g., argon). Transfer of the cryo-cooled specimen puck into the LEAP is enabled via a docking flange (Fig. 1d) that leads to a carousel mounted on a rotatable manipulator that accepts the transferred specimen puck and can be actively cooled via a liquid nitrogen feedthrough when low-temperature operation/transfer is desired. The specimen is then transferred into the LEAP for nanoscale tomographic compositional analysis via the ETH transfer rod shown in Fig. 1b, 2. Note that, for APT analysis, specimens are kept at cryogenic temperatures 25–100 K at all times. For non-cryogenic specimens that are air sensitive and prepared under vacuum within a FIB/SEM, such as nanoscale catalytic metals, environmental transfer under inert or high vacuum is achieved via the ETH following the procedure described above. Such specimens can further be transferred under vacuum into other devices that are attached to the ETH designed to induce morphological and/or chemical changes, such as within the high-temperature ambient pressure reactor shown in Fig. 1a. These samples can then be transferred in situ back into the LEAP for multistage APT analysis using the transfer rod in Fig. 1b, or transferred out via the ETH and transfer shuttle for analysis using other techniques.

To validate the ability of the ETH to facilitate experiments that cannot be performed otherwise, we performed cryogenic APT mass spectral analysis of the organometallic molecule Trimethyl(methylcyclopentadienyl)platinum(IV) (molecular mass 319.3 Da, and abbreviated as MeCpPtMe_3_). The MeCpPtMe_3_ molecule is commonly used within a FIB/SEM to deposit Pt films using Ga ion beam-assisted deposition [12] as a way to protect samples during Ga ion milling. However, the boiling point and melting point prevent ambient transfer of intact films of MeCpPtMe_3_. The conventional preparation of protective films at ambient conditions requires the decomposition of the MeCpPtMe_3_ molecules by exposure to an electron or ion beam to form films consisting of metallic Pt nanoparticles embedded within an amorphous carbonaceous matrix (Pt/a-C) [13]. These qualities make MeCpPtMe_3_ an ideal sample to validate the cryogenic transfer capabilities since the APT spectra would clearly demonstrate whether the film is pristine or suffered contamination or degradation due to thermal warming. If pristine, only peaks corresponding to the fully intact molecule are expected, whereas contamination or degradation would result in both the presence of low molecular weight fragments and isolated platinum ions. Thus, we prepared and analyzed with APT specimens of MeCpPtMe_3_ using either electron beam-assisted deposition (EBAD) or ion beam-assisted deposition (IBAD) at room temperature within a FIB/SEM (conventional approach), and compared those data against cryogenically prepared and cryogenically transferred pristine films.

Specimens of Pt/a-C were prepared from MeCpPtMe_3_ at room temperature using EBAD or IBAD and transferred under ambient conditions into the LEAP. A qualitatively similar time-of-flight (ToF) mass spectrum is observed for IBAD prepared specimens analyzed with APT using both voltage-pulsing and laser-pulsing modes shown in Fig. 2a, b, respectively. Note that the combination of a high-pulsed laser frequency during data collection of the mass spectrum shown in Fig. 2a resulted in a mass cutoff below 250 Da. Therefore, to avoid concerns over whether any laser-pulsing mode would thermally modify any cryogenic samples and affect interpretation, we compare only voltage-pulsed mode for the remaining experiments. The APT ToF mass spectra collected under voltage-pulsing mode are also qualitatively similar for both the IBAD (Fig. 2b) and the EBAD (Fig. 2c) specimens, although the EBAD specimens ran slightly longer before fracture. All IBAD and EBAD specimens showed clear mass peaks of elemental carbon and complex carbon molecules as well as Pt in the 1+ charge state centered at 195 Da and 2+ charge state centered at 97.5 Da (Fig. 2e–g). However, no intact MeCpPtMe_3_^+^ ions at 319.3 Da are seen. All of these results are consistent with the observation that MeCpPtMe_3_ is decomposed to Pt/a-C by electron and ion beams during IBAD and EBAD depositions at room temperature and transfer under ambient conditions.

In comparison, the cryogenic preparation and controlled environmental transfer of MeCpPtMe_3_ lead to the APT ToF detection of only intact MeCpPtMe_3_ molecules. The APT time-of-flight mass spectrum collected under voltage pulsing mode of MeCpPtMe_3_ that has been cryogenically deposited as a thin film within the FIB/SEM and cryogenically transferred to the LEAP using the ETH, shows only a single mass peak group centered at 319 Da (Fig. 2d), the mass of which is consistent with the intact MeCpPtMe_3_^+^ molecule (C_9_H_16_Pt = 319 Da). Zooming-in on the region around 319 Da as shown in Fig. 2h clearly identifies the individual isotopes of the MeCpPtMe_3_ molecule (derived from natural isotopes of Pt). Besides not showing any evidence of individual atoms of elemental Pt^+^ or Pt^2+^ the cryogenically transferred spectra also lacks any detection of elemental carbon and complex carbon ions as seen with IBAD and EBAD specimens (Fig. 3). The absence of other mass peaks within the analyzed volume indicates that the specimen was composed of pristine MeCpPtMe_3_ and that the specimen was fully protected from unwanted environmental exposure during the environmental transfer process.

The ability to prepare and transfer specimens in precise environments provides a means to access new science across many disciplines. In particular, major gains are expected in biology and materials science. In biology, we anticipate that site-specific targeting with cryogenic APT could empower quantification of cellular heterogeneity such as molecular or metal-ion gradients between organelles or buried biointerfaces with part-per-million sensitivity which is currently inaccessible by other means. In materials science, environmental transfer would remove ambiguity in the analysis of easily oxidized or corroded specimens such as lithium-ion battery materials or allow direct time-resolved in situ analysis of chemical and morphological catalyst transformations.

## Methods

### Preparation of Pt/C films at room temperature from MeCpPtMe_3_ for APT analysis

A FEI Helios Nanolab 600 FIB/SEM was used to image and prepare specimens. Circular pads of Pt/a-C with dimensions 1.5 µm diameter by about 600 nm thick were deposited at room temperature onto the top of individual Si posts of a micropost array coupon (Cameca Instruments Inc.–APT division, PN 23265) using either electron beam-assisted deposition (5 kV, 0.17 nA) or ion beam-assisted deposition (30 kV, 90 pA). A sharp needle-like morphology with end radius of <100 nm was made using 30 kV Ga ion milling in an annular pattern oriented along the long axis of the post. Specimens were then transferred under ambient conditions into the LEAP for analysis via the standard specimen load lock.

### Cryo-preparation of MeCpPtMe_3_ for APT analysis

A FEI Helios Nanolab 600 FIB/SEM was used to image and cryogenically prepare specimens. Cryogenic high-vacuum transfer into and out of the FIB/SEM was performed via a Quorum PT2010 cryo-preparation station using the modified specimen transfer shuttle device shown in Fig. 1e. A silicon micropost array substrate (Cameca Instruments Inc.–APT division, PN 23265) attached to the LEAP-compatible specimen puck was loaded into the manipulator of the transfer shuttle and plunged into a liquid nitrogen (lN_2_) slush bath of the Quorum work station. The frozen specimen substrate was then retracted into the specimen isolation chamber and transferred directly onto the cryo-stage within the FIB/SEM. The FIB/SEM stage was precooled to 113 K or colder prior to the transfer into the FIB/SEM. In order to deposit a solid film of MeCpPtMe_3_, was introduced as a vapor via the integrated gas injection system (GIS), which is a common accessory with FEI-brand FIB/SEM systems. The deposition of the MeCpPtMe_3_ resulted in a thick over coating. To prepare a sharp needle-like morphology with end radius of <100 nm, 30 kV Ga ion milling in an annular pattern oriented along the long axis of the post was performed. The substrate was then transferred out of the FIB/SEM using the customized specimen transfer shuttle device and loaded onto the liquid nitrogen-cooled specimen carousel of the ETH (Fig. 1d). The cryo-prepared specimen was then transferred into the LEAP for analysis. Note that the receiving specimen stage within the LEAP analysis chamber was precooled and held at a temperature around 40 K for APT analysis.

### APT analysis

APT analysis was performed using a LEAP 4000×-HR from Cameca Instruments. Voltage pulsing mode was applied at a frequency of 125 kHz and a 10% pulse fraction. For the pulsed laser APT experiments, a laser energy of 160–180 pJ was used at frequency of 125 kHz. A detection rate of about 200–400 ions/s, stage temperature of 40–50 K, and analysis chamber pressure of <4 × 10^−9^ Pa were maintained for the duration of all experiments.

## Abbreviations

3D: three-dimensional
APT: atom probe tomography
EBAD: electron beam-assisted deposition
ETH: environmental transfer hub
FIB/SEM: focused ion beam/scanning electron microscope
GIS: gas injection system
IBAD: ion beam-assisted deposition
LEAP: local electrode atom probe
MeCpPtMe_3_: trimethyl(methylcyclopentadienyl)platinum(IV)
Pt/a-C: Pt nanoparticles embedded within an amorphous carbonaceous matrix
ToF: time of flight
UHV: ultrahigh vacuum
